# Crystal structure of (4-cyano­pyridine-κ*N*){5,10,15,20-tetrakis[4-(benzoyloxy)phenyl]porphyrinato-κ^4^ *N*}zinc–4-cyano­pyridine (1/1)

**DOI:** 10.1107/S2056989016000062

**Published:** 2016-01-13

**Authors:** Soumaya Nasri, Nesrine Amiri, Ilona Turowska-Tyrk, Jean-Claude Daran, Habib Nasri

**Affiliations:** a Laboratoire de Physico-chimie des Matériaux, Faculté des Sciences de Monastir, Avenue de l’environnement, 5019 Monastir, University of Monastir, Tunisia; b Faculty of Chemistry, Wroław University of Technology, Wybrzeże Wyspiańskiego 27, 50-370 Wroław, Poland; c Laboratoire de Chimie de Coordination, CNRS UPR 8241, 205 route de Norbonne, 31077 Toulouse, Cedex 04, France

**Keywords:** crystal structure, zinc porphyrin, 4-cyano­pyridine, hydrogen bonds, FT–IR

## Abstract

In the title compound, [Zn(C_72_H_44_N_4_O_8_)(C_6_H_4_N_2_)]·C_6_H_4_N_2_ or [Zn(TPBP)(4-CNpy]·(4-CNpy) [where TPBP and 4-CNpy are 5,10,15,20-(tetra­phenyl­benzoate)porphyrinate and 4-cyano­pyridine, respectively], the Zn^II^ cation is chelated by four pyrrole-N atoms of the porphyrinate anion and coordinated by a pyridyl-N atom of the 4-CNpy axial ligand in a distorted square-pyramidal geometry. The average Zn—N(pyrrole) bond length is 2.060 (6) Å and the Zn—N(4-CNpy) bond length is 2.159 (2) Å. The zinc cation is displaced by 0.319 (1) Å from the N_4_C_20_ mean plane of the porphyrinate anion toward the 4-cyano­pyridine axial ligand. This porphyrinate macrocycle exhibits major saddle and moderate ruffling and doming deformations. In the crystal, the [Zn(TPBP)(4-CNpy)] complex mol­ecules are linked together *via* weak C—H⋯N, C—H⋯O and C—H⋯π inter­actions, forming supra­molecular channels parallel to the *c* axis. The non-coordinating 4-cyano­pyridine mol­ecules are located in the channels and linked with the complex mol­ecules, *via* weak C—H⋯N inter­actions and π-π stacking or *via* weak C—H⋯O and C—H⋯π inter­actions. The non-coordinating 4-cyano­pyridine mol­ecule is disordered over two positions with an occupancy ratio of 0.666 (4):0.334 (4).

## Abstract 2

In the crystal, the Zn^II^ cation is chelated by four pyrrole-N atoms of the porphyrinate anion and coordinated by a pyridyl-N atom of the 4-cyano­pyridine axial ligand in a distorted square-pyramidal geometry. The non-coordinating 4-cyano­pyridine mol­ecule is disordered over two positions in the supra­molecular channel formed by complex mol­ecules.

## Chemical context   

During the last two decades, renewed attention to zinc metalloporphyrins has been noted for their applications in different fields *e.g.* solar energy harvesting and artificial photosynthesis (Aratani *et al.*, 2009 ▸; Panda *et al.*, 2012 ▸) and as building blocks of assemblies (Diskin-Posner *et al.*, 2002 ▸). Many structures of five-coordinate zinc porphyrins of the type [Zn(Porph)(*L*)] (Porph = is a porphyrinato ligand and *L* is a neutral unidentate ligand N-bonded to the zinc cation) are known in the literature. However, only three structures of zinc–4-NCpy non-porphyrinic species [CSD refcodes CYPYZN (Steffen & Palenik, 1977 ▸); LIMWUZ (Clegg *et al.*, 1995 ▸) and QIDXAD (Huang *et al.*, 2007 ▸; CCD Version 5.35 (Groom & Allen, 2014 ▸)] and one structure of a zinc–4-NCpy-porphyrin derivative are reported in the literature (CSD refcode IRAFIR; Brahma *et al.*, 2011 ▸). To gain more insight into the structural and spectroscopic properties of Zn^II^–N-donor monodentate neutral ligand metalloporphyrins in general and Zn^II^-cyano­pyridine porphyrin derivatives in particular, we report herein the synthesis, the mol­ecular structure and the spectroscopic data of the title compound with the formula [Zn(TPBP)(4-CNpy)]·(4-CNpy) (I).
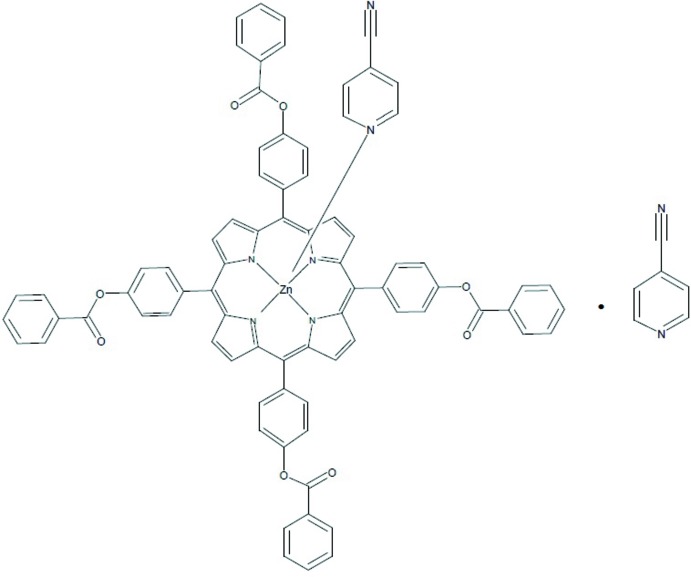


## Structural commentary   

**Figure 1:**
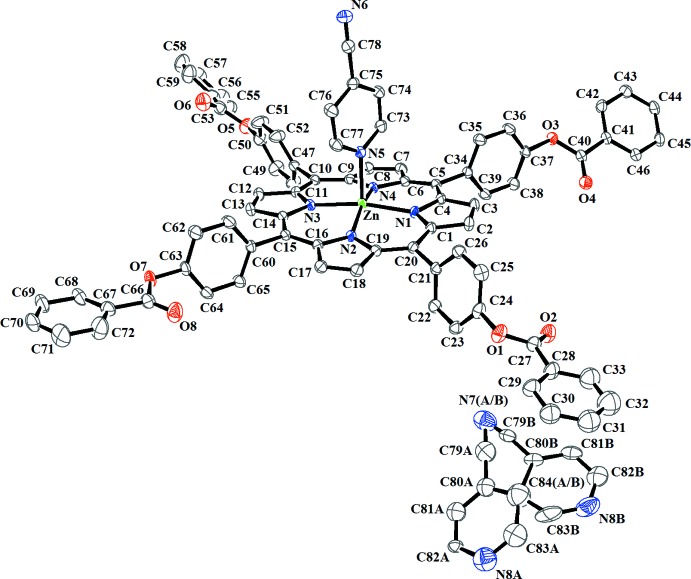
An *ORTEP* view of the mol­ecular structure of the title compound with the atom-numbering scheme. Displacement ellipsoids are drawn at the 40% probability level. H atoms have been omitted for clarity.

**Figure 2:**
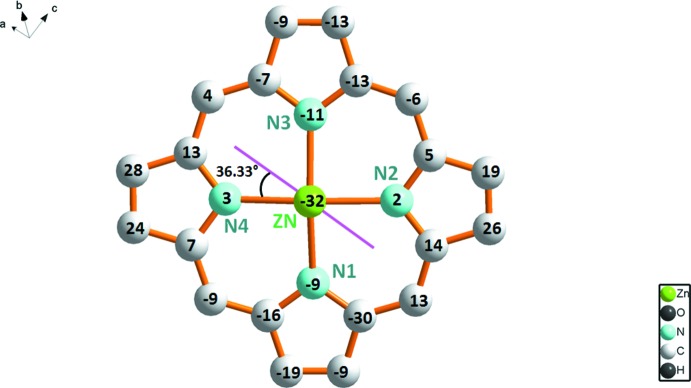
Formal diagram of the porphyrinate core illustrating the displacements of each atom from the 24-atoms core plane in units of 0.01 Å.

The central Zn^II^ cation of the [Zn(TPBP)(4-CNpy)] complex has a distorted square-pyramidal coordination geometry (Fig. 1 ▸). The equatorial plane is formed by four nitro­gen atoms of the porphyrin whereas the apical position is occupied by the 4-cyano­pyridine ligand. The asymmetric unit of (I) consists of the [Zn(TPBP)(4-CNpy)] complex and one 4-cyano­pyridine mol­ecule. The Zn^__^N(4-CNpy) bond length [2.159 (2) Å] is in the range (2.055–2.248 Å) of those of the zinc–4-CNpy complexes reported in the literature [CSD refcodes LIMWUZ (Clegg *et al.* 1995 ▸) and QIDXAD (Huang *et al.*, 2007 ▸)]. The average equatorial zinc–N(pyrrole) distance (Zn—Np) is 2.060 (6) Å which is close to those in related zinc metalloporphyrins of type [Zn(Porph)(*L*)] (Porph and *L* are a porphyrinato and a monodentate neutral ligand, respectively) [CSD refcodes ATUSOX (Vinodu & Goldberg, 2004 ▸) and GEPBAF (Lipstman *et al.*, 2006 ▸)]. A formal diagram of the porphyrinato cores of (I) showing the displacements of each atom from the mean plane of the 24-atom porphyrin macrocycle in units of 0.01 Å is illustrated in Fig. 2 ▸. The zinc atom is displaced by 0.319 (1) Å from the 24-atom porphyrin mean plane (P_C_). This value is close to those of the related five-coordinated zinc metalloporphyrins [Zn(TPP)(DMSO)] (DMSO = dimethyl sulfoxide, Zn—P_C_ = 0.338 Å; Vinodu & Goldberg, 2004 ▸) and [Zn(TPP)(DMAC)] (DMAC = *N*,*N*-di­methyl­acetamide, Zn—P_C_ = 0.377 Å; Lipstman *et al.*, 2006 ▸). The porphyrin core presents a major *saddle* and a moderate *ruffling* and *doming* distortion (Scheidt & Lee, 1987 ▸).

The *saddle* deformation is due to the displacement of the pyrrole rings alternately above and below the mean porphyrin macrocycle so that the pyrrole nitro­gen atoms are out of the mean plane. The *ruffling* distortion is indicated by the high values of the displacement of the *meso*-carbon atoms above and below the porphyrin mean plane while the *doming* deformation is originated by the displacement of the metal atom out of the mean plane, and the nitro­gen atoms are displaced toward the axial ligand. Generally, for hemoproteins and metalloporphyrins, the plane of the axial ligand (*i.e*., imidazole, pyridine) nearly bis­ects the ‘*cis*’ Np—Fe—Np angle, which is also the case for the title zinc–4-CNpy deriv­ative (I) where the dihedral angle between the plane of the 4-CNpy ligand and the N4–Zn–N5 plane is 36.33 (12)° (Fig. 2 ▸).

## Supra­molecular features   

**Figure 3:**
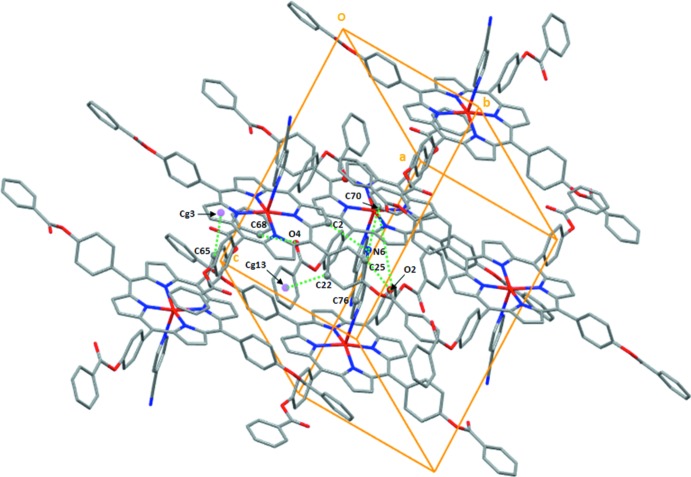
A partial view of the crystal packing of (I) showing the link between the [Zn(TPBP)(4-cyano)] complexes *via* non-classical C—H⋯N and C—H⋯O hydrogen bonds and by C—H⋯π inter­actions. The non-coordinating 4-cyano­pyridine mol­ecules are omitted for clarity.

Within the crystal structure of (I) (Fig. 3 ▸), the [Zn(TPBP)(4-CNpy)] complexes are linked together *via* weak non-classical C—H⋯N and C—H⋯O hydrogen bonds and by C—H⋯π inter­actions (Table 1 ▸). The nitro­gen atom N6 of the cyano group of the 4-CNpy axial ligand is involved in C—H⋯N hydrogen bonding and short contact inter­actions with the carbon atoms C2, C25 and C70 of the nearby [Zn(TPBP)(4-CNpy)] complexes with C—H⋯N6 distances of 3.284 (4), 3.393 (4) and 3.246 (6) Å, respectively. The oxygen atom O2 of the carbonyl group of one arm of one TPBP porphyrinato ligand inter­acts with the carbon atom C25 of a phenyl ring of an adjacent porphyrin [C25⋯O2 = 3.524 (4) Å] and the carbon atom C76 of the closest [Zn(TPBP)(4-CNpy)] complex [C76⋯O2 = 3.174 (4) Å]. The oxygen atom O4 of a carbonyl group of a second arm of the TPBP porphyrinato ligand is weakly linked to the carbon atom C68 of a phenyl ring of an adjacent TPBP porphyrinato ligand [C68—HC8⋯O4 distance = 3.150 (4) Å]. These [Zn(TPBP)(4-CNpy)] complexes are also linked by weak C—H⋯*Cg* intra­molecular inter­actions involving the carbon atoms C22 and C65 of a phenyl rings of two TPBP porphyrinato ligands and the centroids *Cg*13 and *Cg*3 of the pyrrole rings of two adjacent porphyrins. The values of these C—H⋯*Cg* inter­actions are 3.650 (3) Å and 3.457 (4) Å, respectively.

**Figure 4:**
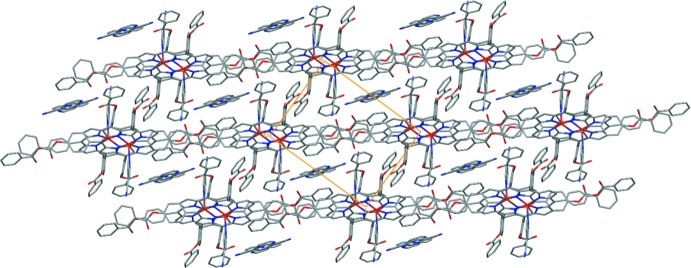
The crystal structure of the title compound plotted in projection along [001] showing the disordered non-coordinating 4-cyano­pyridine mol­ecules occupying the channels between the [Zn(TPBP)(4-CNpy)] complex mol­ecules. H atoms have been omitted.

**Figure 5:**
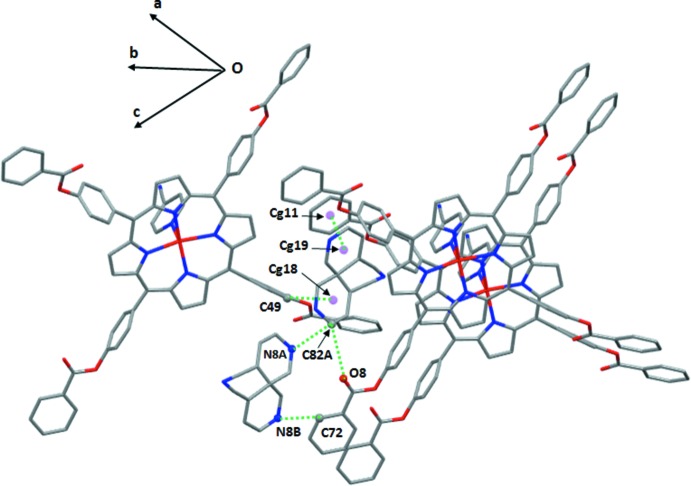
Drawing showing the C—H⋯N and C—H⋯O hydrogen bonds and the C—H⋯π inter­actions between a disordered non-coordinating 4-cyano­pyridine mol­ecule and a neighboring [Zn(TPBP)(4-CNpy)] complex and a free 4-cyano­pyridine mol­ecule.

It is noteworthy that the non-coordinating 4-CNpy mol­ecules are located in the channels between the [Zn(TPBP)(4-CNpy)] complexes parallel to the *c* axis (Fig. 4 ▸). Each free disordered 4-cyano­pyridine mol­ecule is linked to three adjacent [Zn(TPBP)(4-CNpy)] complexes *via* (i) atom C82*A* of the free 4-NCpy mol­ecule and atom O8 of a TPBP porphyrin [C82*A*—H82*A*⋯O8 distance = 3.226 (5) Å], (ii) the centroid (*Cg*18) of the C80*A*–C81*A*–C82*A*–N8*A*–C83*A*–C84*A* ring of the disordered free 4-CNpy mol­ecule and the carbon atom C49 of an adjacent TPBR porphyrinato ligand with a C49—H49⋯*Cg*18 contact length of 3.448 (4) Å, (iii) by aromatic π–π inter­actions between the centroid (*Cg*19) of the C80*A*–C81*B*–C82*B*–N8*B*–C83*B*–C84*B* ring of a free disordered 4-CNpy mol­ecule and the centroid (*Cg*11) of the phenyl porphyrin ring C28–C33 [*Cg*19⋯*Cg*11 = 3.668 (4) Å; Table 2 ▸). On the other hand, the C82*A* carbon atom of one disordered 4-cyano­pyridine mol­ecule is also weakly linked to the nitro­gen atom N8*A* of a second 4-CNpy free mol­ecule [C82*A*—H82*A*⋯N8*A* distance = 2.934 (8) Å] and the N8*B* nitro­gen atom of this second 4-CNpy mol­ecule is weakly bonded to the carbon atom C72 of a phenyl ring of a nearby TPBR porphyrinato ligand [C72^__^H72⋯N8*B* distance = 3.226 (15) Å] (Fig. 5 ▸).

### Synthesis and crystallization   

4-Formyl­phenyl­ester was prepared from benzoic acid and 4-hy­droxy­benzaldehyde. 5,10,15,20-tetra­phenyl­benzo­ate­porphyrin (H_2_TPBP) and the starting [Zn(TPBP)] complex were synthesized using modified reported methods (Adler *et al.*, 1967 ▸; Oberda *et al.*, 2011 ▸). The title complex (I) was made by reaction of the [Zn(TPBP)] complex with an excess of 4-cyano­pyridine in di­chloro­methane at room temperature.

### Synthesis of 4-formyl­phenyl­benzoate   

Benzoic acid (6 g, 0.049 mol), 4-hy­droxy­benzaldehyde (6 g, 0,049 mol) and di­methyl­amino­pyridin DMAP (0.6 g, 0.0049) were dissolved at 273 K in 20 mL of di­chloro­methane. To this solution, 10.12 g of *N*,*N*′-di­cyclo­hexyl­carbodi­imide DCC (0.049 mol) dissolved in 33 mL of di­chloro­methane was added dropwise and stirred at 273 K and then at room temperature for 12 h. Upon completion, the reaction mixture was filtered and the solvent was evaporated to dryness, to afford 9.3 g of a pale-yellow solid (yield 86%), m.p. = 356–358 K, C_14_H_10_O_3_: C 74.33, H 4.46%; found: C 73.98, H 4.35%. Spectroscopic analysis: ^1^H NMR (300 MHz, DMSO-*d*_6_) δ_H_ (p.p.m.) 10.04 (*s*, 1H), 8.17 (*d*, 2H, *J* = 6 Hz), 8.04 (*d*, 2H, *J* = 9 Hz), 7.80 (*m*, 1H), 7.64 (*m*, 2H), 7.56 (*d*, 2H, *J* = 9 Hz). ^13^C NMR (75 MHz, DMSO-*d*_6_) δ_C_ (p.p.m.) 192.09, 164.12, 155.21, 134.31, 134, 131.13, 129.91, 129.03, 128.47, 122.90.

### Synthesis of 5,10,15,20-(tetra­phenyl­benzoate)porphyrin   

4.5 mg of 4-formyl­phenyl­benzoate (19.9 mmol) were dissolved in 50 mL of propionic acid. The solution was heated under reflex at 413 K. Freshly distilled pyrrole (1.4 mL, 19.9 mmol) was then added dropwise and the mixture was stirred for another 40 min. The mixture was cooled overnight at 277 K and filtered under vacuum. The crude product was purified using column chromatography (chloro­form/petroleum ether 4/1 *v*/*v* as an eluent). A purple solid was obtained and dried under vacuum (1.18 g, yield 21%).

Spectroscopic analysis: ^1^H NMR (300 MHz, CDCl_3_) δ (p.p.m.) 8.94 (*S*, 8H), 8.39 (*d*, 8H, *J* = 6 Hz), 8.29 (*d*, 8H, *J* = 9 Hz), 7.71 (*S*, 8H), 7.62 (*m*, 12H), −2.80 (*S*, 2H). UV/Vis (CHCl_3_): λ_max_ (10^−3^ ∊, mol^−1^ l^−1^ cm^−1^) 420 (512.7), 516 (16.7), 552 (7.4), 591 (4.8), 646 (4.0).

### Synthesis of [5,10,15,20-(tetra­phenyl­benzoate)porphyrinato]zinc(II)   

A mixture of the H_2_TPBP porphyrin (400 mg, 0.365 mmol) and [Zn(OAc)_2_]·2H_2_O (700 mg, 3.650 mmol) in CHCl_3_ (30 mL) and CH_3_OH (5 mL) was stirred at room temperature overnight. The solvent was evaporated and a light-purple solid of the [Zn(TPBP)] complex was obtained (350 mg, yield 87.5%).

Spectroscopic analysis: ^1^H NMR (300 MHz, CDCl_3_) δ(p.p.m. ) 9.04 (*S*, 8H), 8.40 (*d*, 8H, *J* = 9 Hz), 8.30 (*m*, 8H), 7.85 (*S*, 8H), 7.64 (*m*, 12H), −2.80 (*S*, 2H). UV/Vis (CHCl_3_):λ_max_ (10^−3^ ∊, mol^−1^ l^−1^ cm^−1^) (10^−3^ ∊) 425 (613.5), 554 (23.0), 596 (6.9).

### Synthesis and crystallization of the title complex (I)   

To a solution of [Zn(TPBP)] (100 mg, 0.086 mmol) in di­chloro­methane (5 mL) was added an excess of 4-cyano­pyridine (200 mg, 0.192 mmol). The reaction mixture was stirred at room temperature for 2 h. Single crystals of the title complex were obtained by diffusion of hexa­nes through the di­chloro­methane solution.

Spectroscopic analysis: ^1^H NMR (300 MHz, CDCl_3_) δ(p.p.m. ) 9.04 (*S*, 8H), 8.40 (*d*, 8H, *J* = 7.5 Hz), 8.30 (*d*, 8H, *J* = 9 Hz), 7.67 (*m*, 20H), 7.53 (*m*, 2H). UV/Vis (CHCl_3_): λ_max_ (10^−3^ ∊, mol^−1^ l^−1^ cm^−1^) 425 (613.5), 554 (23.0), 596 (6.9).

## FT–IR spectroscopy   

**Figure 6:**
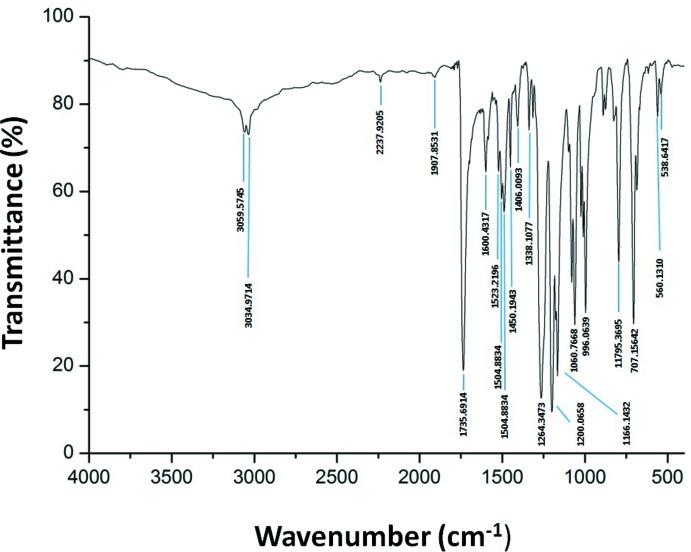
FT–IR spectrum of (I).

The FT–IR spectrum of [Zn(TPBP)(4-CNpy)]·(4-CNpy) (I) (Fig. 6 ▸) was recorded in the 4000–400 cm^−1^ domain using a Perkin–Elmer Spectrum Two FTIR spectrometer. The spectrum presents characteristic IR bands of the TPBP porphyrinato moiety. The C—H stretching frequencies of the porphyrin are in the range 3060–2860 cm^−1^, the ester group of the *meso*-substituents of this porphyrin are identified by a strong band at 1736 cm^−1^, ν(C=O) stretch and by two strong bands at 1264 and 1061 corresponding to the ν(C—O) stretching vibration. The IR spectrum of (I) also shows a very weak absorption band at 2238 cm^−1^ attributed to the nitrile stretching frequency ν(C N). The value of this band is almost identical to the one of the free 4-cyano­pyridine (2236 cm^−1^) which could be attributed both to the 4-CNpy ligand or the free 4-CNpy mol­ecule in (I) because this band is usually not affected by the coordination of the 4-cyano­pyridine (Singh *et al.*, 2000 ▸). On the other hand, the IR spectrum of the title compound exhibits several absorption bands at 1907 cm^−1^ (*vw*: very weak), 1523 cm^−1^ (*vw*), 1505 cm^−1^(*w*: weak), 1406 cm^−1^ (*m*: medium), 996 cm^−1^ (*s*: strong), 707 cm^−1^ (*m*), 685 cm^−1^ (*m*) and 538 cm^−1^ (*w*) attributed to the pyridyl group of the coordinating and the free 4-cyano­pyridine species (Singh *et al.*, 2000 ▸).

## Refinement details   

Crystal data, data collection and structure refinement details are summarized in Table 3 ▸. Hydrogen atoms were placed in calculated positions and refined as riding atoms: C—H = 0.92 Å with *U*_iso_(H) = 1.2 *U*_eq_(C). The non-coordinating 4-cyano­pyridine mol­ecule is disordered over two positions *A* and *B* with refined occupancies of 0.666 (4) and 0.334 (4), respectively. The bond lengths and angles of this mol­ecule were restrained to ensure proper geometry using DFIX and DANG instructions of *SHELXL2014* (Sheldrick, 2015 ▸). The anisotropic displacement ellipsoids of some atoms of the disordered 4-cyano­pyridine free mol­ecule were very elongated which indicates static disorder. For these atoms, SIMU/ISOR restraints were applied (McArdle, 1995 ▸; Sheldrick, 2008 ▸).

## Supplementary Material

Crystal structure: contains datablock(s) I, global. DOI: 10.1107/S2056989016000062/xu5882sup1.cif

Structure factors: contains datablock(s) I. DOI: 10.1107/S2056989016000062/xu5882Isup2.hkl

CCDC reference: 1445100

Additional supporting information:  crystallographic information; 3D view; checkCIF report

## Figures and Tables

**Table 1:** Hydrogen-bond geometry (Å, °) *Cg*3, *Cg*13, *Cg*18 are the centroids of the N3/C11–C14, C41–C46 and N8*A*–C82*A*–C81*A*–C80*A*–C84*A*–C83*A* rings, respectively.

*D*—H⋯*A*	*D*—H	H⋯*A*	*D*⋯*A*	*D*—H⋯*A*
C2—H2⋯N6^i^	0.93	2.45	3.284 (4)	149
C25—H25⋯N6^ii^	0.93	2.52	3.393 (4)	157
C68—H68⋯O4^iii^	0.93	2.41	3.150 (4)	136
C72—H72⋯N8*B* ^iv^	0.93	2.58	3.226 (15)	127
C82*A*—H82*A*⋯O8^i^	0.93	2.38	3.226 (5)	152
C22—H22⋯*Cg*13^v^	0.93	2.82	3.650 (3)	150
C49—H49⋯*Cg*18^vi^	0.93	2.61	3.448 (4)	151
C65—H65⋯*Cg*3^vii^	0.93	2.65	3.457 (4)	145

Symmetry codes: (i) 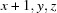; (ii) 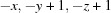; (iii) 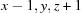; (iv) 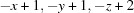; (v) 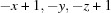; (vi) 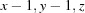; (vii) 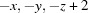.

**Table 2:** π–π inter­actions (Å, °) *Cg*⋯*Cg* = distance between ring centroids, α = dihedral angle between planes *I* and *J*, *CgI*_Perp = perpendicular distance of *Cg*(I) on ring *J*, *CgJ*_Perp = perpendicular distance of *Cg*(J) on ring *I. *Cg**(11) and *Cg*(19) are the centroids of C28–C33 and N8*B*–C82*B*–C81*B*–C80*B*–C84*B*–C83*B* rings, respectively.

*Cg*(*I*)	*Cg*(*J*)	*Cg*⋯*Cg*	α	*CgI*_Perp	*CgJ*_Perp
*Cg*(11)	*Cg*(19)^i^	3.668 (4)	19.1 (4)	3.601 (4)	3.366 (2)

Symmetry code: (i) *x*, *y*, *z*.

**Table 3:** Experimental details

Crystal data	
Chemical formula	[Zn(C_72_H_44_N_4_O_8_)(C_6_H_4_N_2_)]·C_6_H_4_N_2_
*M*_r_	1366.70
Crystal system, space group	Triclinic, *P* 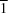
Temperature (K)	100
*a*, *b*, *c* (Å)	11.8587 (3), 16.1619 (5), 19.2167 (5)
α, β, γ (°)	68.207 (3), 81.077 (2), 86.866 (2)
*V* (Å^3^)	3378.43 (18)
*Z*	2
Radiation type	Mo *K*α
μ (mm^−1^)	0.43
Crystal size (mm)	0.38 × 0.13 × 0.07

Data collection	
Diffractometer	Agilent Xcalibur, Eos, Gemini ultra
Absorption correction	Multi-scan (*CrysAlis PRO*; Agilent, 2014 ▸)
*T*_min_, *T*_max_	0.830, 1.000
No. of measured, independent and observed [*I* > 2σ(*I*)] reflections	37755, 15805, 11876
*R*_int_	0.029
(sin θ/λ)_max_ (Å^−1^)	0.693

Refinement	
*R*[*F* ^2^ > 2σ(*F* ^2^)], *wR*(*F* ^2^), *S*	0.054, 0.155, 1.02
No. of reflections	15805
No. of parameters	965
No. of restraints	138
H-atom treatment	H-atom parameters constrained
Δρ_max_, Δρ_min_ (e Å^−3^)	1.17, −0.86

Computer programs: *CrysAlis PRO* (Agilent, 2014 ▸), *SIR2004* (Burla *et al.*, 2005 ▸), *SHELXL2013* (Sheldrick, 2015 ▸), *ORTEPIII* (Burnett & Johnson, 1996 ▸) and *WinGX* publication routines (Farrugia, 2012 ▸).

## supplementary crystallographic information

### Crystal data

**Table:**

C_78_H_48_N_6_O_8_Zn·C_6_H_4_N_2_	*Z* = 2
*M**_r_* = 1366.70	*F*(000) = 1412
Triclinic, *P*1	*D*_x_ = 1.344 Mg m^−^^3^*D*_m_ = 1.344 Mg m^−^^3^*D*_m_ measured by ?
*a* = 11.8587 (3) Å	Mo *K*α radiation, λ = 0.71073 Å
*b* = 16.1619 (5) Å	Cell parameters from 9704 reflections
*c* = 19.2167 (5) Å	θ = 3.8–28.8°
α = 68.207 (3)°	µ = 0.43 mm^−^^1^
β = 81.077 (2)°	*T* = 100 K
γ = 86.866 (2)°	Block, purple
*V* = 3378.43 (18) Å^3^	0.38 × 0.13 × 0.07 mm

### Data collection

**Table:**

Agilent Xcalibur, Eos, Gemini ultra diffractometer	15805 independent reflections
Radiation source: fine-focus sealed tube	11876 reflections with *I* > 2σ(*I*)
Detector resolution: 16.1978 pixels mm^-1^	*R*_int_ = 0.029
ω scans	θ_max_ = 29.5°, θ_min_ = 3.0°
Absorption correction: multi-scan *CrysAlis PRO* (Agilent, 2014)	*h* = −15→16
*T*_min_ = 0.830, *T*_max_ = 1.000	*k* = −22→19
37755 measured reflections	*l* = −24→25

### Refinement

**Table:**

Refinement on *F*^2^	138 restraints
Least-squares matrix: full	Hydrogen site location: inferred from neighbouring sites
*R*[*F*^2^ > 2σ(*F*^2^)] = 0.054	H-atom parameters constrained
*wR*(*F*^2^) = 0.155	*w* = 1/[σ^2^(*F*_o_^2^) + (0.0732*P*)^2^ + 3.5911*P*] where *P* = (*F*_o_^2^ + 2*F*_c_^2^)/3
*S* = 1.02	(Δ/σ)_max_ = 0.001
15805 reflections	Δρ_max_ = 1.17 e Å^−^^3^
965 parameters	Δρ_min_ = −0.86 e Å^−^^3^

### Special details

**Table:**

Geometry. All e.s.d.'s (except the e.s.d. in the dihedral angle between two l.s. planes) are estimated using the full covariance matrix. The cell e.s.d.'s are taken into account individually in the estimation of e.s.d.'s in distances, angles and torsion angles; correlations between e.s.d.'s in cell parameters are only used when they are defined by crystal symmetry. An approximate (isotropic) treatment of cell e.s.d.'s is used for estimating e.s.d.'s involving l.s. planes.

### Fractional atomic coordinates and isotropic or equivalent isotropic displacement parameters (Å^2^)

**Table:**

	*x*	*y*	*z*	*U*_iso_*/*U*_eq_	Occ. (<1)
Zn	0.04026 (2)	0.08329 (2)	0.73701 (2)	0.02122 (9)	
O1	0.57584 (18)	0.51097 (14)	0.64208 (13)	0.0426 (5)	
O2	0.70068 (19)	0.45789 (15)	0.56891 (13)	0.0458 (5)	
O3	0.37839 (16)	−0.04282 (14)	0.32980 (10)	0.0327 (4)	
O4	0.55891 (18)	−0.00492 (15)	0.32873 (11)	0.0406 (5)	
O5	−0.3951 (2)	−0.41779 (15)	0.87976 (14)	0.0508 (6)	
O6	−0.5645 (2)	−0.36754 (16)	0.91940 (14)	0.0546 (6)	
O7	−0.29520 (17)	0.18935 (13)	1.15595 (10)	0.0351 (4)	
O8	−0.1743 (2)	0.29908 (18)	1.14029 (15)	0.0570 (7)	
N1	0.18054 (17)	0.13129 (14)	0.65650 (11)	0.0232 (4)	
N2	0.07731 (17)	0.16791 (14)	0.78840 (11)	0.0240 (4)	
N3	−0.07552 (17)	0.01808 (14)	0.83124 (11)	0.0235 (4)	
N4	0.04160 (17)	−0.02765 (14)	0.70765 (11)	0.0229 (4)	
N5	−0.08471 (18)	0.16108 (14)	0.66898 (11)	0.0252 (4)	
N6	−0.4242 (2)	0.31984 (17)	0.51515 (14)	0.0371 (5)	
C1	0.2447 (2)	0.20434 (17)	0.64469 (13)	0.0240 (5)	
C2	0.3233 (2)	0.22452 (18)	0.57502 (14)	0.0288 (6)	
H2	0.3762	0.2708	0.5543	0.035*	
C3	0.3049 (2)	0.16325 (18)	0.54583 (14)	0.0285 (5)	
H3	0.3421	0.1600	0.5006	0.034*	
C4	0.2173 (2)	0.10365 (17)	0.59760 (13)	0.0235 (5)	
C5	0.1830 (2)	0.02547 (17)	0.59184 (13)	0.0236 (5)	
C6	0.1054 (2)	−0.03754 (16)	0.64541 (13)	0.0234 (5)	
C7	0.0774 (2)	−0.12123 (18)	0.64126 (15)	0.0294 (6)	
H7	0.1094	−0.1438	0.6046	0.035*	
C8	−0.0041 (2)	−0.16061 (18)	0.70068 (15)	0.0294 (5)	
H8	−0.0383	−0.2156	0.7129	0.035*	
C9	−0.0282 (2)	−0.10107 (17)	0.74167 (13)	0.0240 (5)	
C10	−0.1130 (2)	−0.11536 (17)	0.80477 (14)	0.0255 (5)	
C11	−0.1338 (2)	−0.05862 (17)	0.84584 (13)	0.0249 (5)	
C12	−0.2173 (2)	−0.07594 (19)	0.91324 (14)	0.0307 (6)	
H12	−0.2684	−0.1235	0.9348	0.037*	
C13	−0.2066 (2)	−0.00937 (18)	0.93860 (14)	0.0299 (6)	
H13	−0.2490	−0.0027	0.9811	0.036*	
C14	−0.1174 (2)	0.04907 (17)	0.88764 (13)	0.0238 (5)	
C15	−0.0754 (2)	0.12266 (17)	0.89817 (13)	0.0242 (5)	
C16	0.0177 (2)	0.17572 (18)	0.85287 (14)	0.0255 (5)	
C17	0.0644 (2)	0.24837 (19)	0.86626 (15)	0.0307 (6)	
H17	0.0391	0.2674	0.9061	0.037*	
C18	0.1514 (2)	0.28331 (19)	0.80994 (15)	0.0303 (6)	
H18	0.1976	0.3310	0.8037	0.036*	
C19	0.1595 (2)	0.23286 (17)	0.76095 (14)	0.0248 (5)	
C20	0.2387 (2)	0.25056 (16)	0.69438 (14)	0.0243 (5)	
C21	0.3277 (2)	0.32056 (17)	0.67803 (14)	0.0249 (5)	
C22	0.4073 (2)	0.30588 (18)	0.72723 (15)	0.0308 (6)	
H22	0.4044	0.2533	0.7695	0.037*	
C23	0.4911 (2)	0.36854 (19)	0.71428 (16)	0.0335 (6)	
H23	0.5439	0.3586	0.7475	0.040*	
C24	0.4945 (2)	0.44523 (18)	0.65171 (17)	0.0333 (6)	
C25	0.4182 (3)	0.46245 (19)	0.60102 (17)	0.0363 (6)	
H25	0.4228	0.5148	0.5585	0.044*	
C26	0.3339 (2)	0.39930 (18)	0.61516 (16)	0.0320 (6)	
H26	0.2808	0.4100	0.5819	0.038*	
C27	0.6776 (3)	0.50913 (18)	0.60080 (16)	0.0353 (6)	
C28	0.7567 (3)	0.5775 (2)	0.60045 (19)	0.0438 (7)	
C29	0.7336 (3)	0.6249 (2)	0.6476 (2)	0.0480 (8)	
H29	0.6661	0.6147	0.6814	0.058*	
C30	0.8112 (4)	0.6877 (3)	0.6447 (2)	0.0631 (10)	
H30	0.7967	0.7187	0.6774	0.076*	
C31	0.9089 (4)	0.7040 (3)	0.5936 (3)	0.0774 (12)	
H31	0.9603	0.7467	0.5912	0.093*	
C32	0.9322 (5)	0.6576 (4)	0.5454 (3)	0.0913 (15)	
H32	0.9987	0.6694	0.5105	0.110*	
C33	0.8558 (4)	0.5935 (3)	0.5493 (3)	0.0700 (11)	
H33	0.8714	0.5614	0.5175	0.084*	
C34	0.2361 (2)	0.00688 (16)	0.52300 (13)	0.0237 (5)	
C35	0.1878 (2)	0.0403 (2)	0.45691 (15)	0.0334 (6)	
H35	0.1208	0.0732	0.4556	0.040*	
C36	0.2389 (2)	0.0252 (2)	0.39228 (15)	0.0345 (6)	
H36	0.2067	0.0482	0.3478	0.041*	
C37	0.3372 (2)	−0.02394 (17)	0.39509 (14)	0.0264 (5)	
C38	0.3864 (2)	−0.0591 (2)	0.46026 (15)	0.0341 (6)	
H38	0.4523	−0.0933	0.4617	0.041*	
C39	0.3354 (2)	−0.0424 (2)	0.52382 (15)	0.0327 (6)	
H39	0.3688	−0.0647	0.5679	0.039*	
C40	0.4912 (2)	−0.03034 (17)	0.30113 (14)	0.0277 (5)	
C41	0.5181 (2)	−0.05140 (17)	0.23136 (14)	0.0270 (5)	
C42	0.4342 (2)	−0.0700 (2)	0.19637 (15)	0.0335 (6)	
H42	0.3577	−0.0702	0.2165	0.040*	
C43	0.4656 (3)	−0.0883 (2)	0.13095 (16)	0.0386 (7)	
H43	0.4098	−0.1004	0.1069	0.046*	
C44	0.5790 (3)	−0.0888 (2)	0.10143 (15)	0.0363 (6)	
H44	0.5993	−0.1017	0.0579	0.044*	
C45	0.6626 (3)	−0.0702 (2)	0.13595 (15)	0.0372 (6)	
H45	0.7390	−0.0704	0.1156	0.045*	
C46	0.6328 (2)	−0.05105 (19)	0.20101 (14)	0.0318 (6)	
H46	0.6890	−0.0381	0.2243	0.038*	
C47	−0.1892 (2)	−0.19473 (18)	0.82823 (14)	0.0286 (5)	
C48	−0.1499 (3)	−0.2807 (2)	0.85981 (18)	0.0390 (7)	
H48	−0.0744	−0.2903	0.8685	0.047*	
C49	−0.2220 (3)	−0.3531 (2)	0.87866 (19)	0.0438 (7)	
H49	−0.1947	−0.4108	0.8999	0.053*	
C50	−0.3332 (3)	−0.3397 (2)	0.86602 (17)	0.0403 (7)	
C51	−0.3757 (3)	−0.2545 (2)	0.8359 (2)	0.0524 (9)	
H51	−0.4516	−0.2453	0.8281	0.063*	
C52	−0.3028 (3)	−0.1831 (2)	0.81779 (18)	0.0443 (8)	
H52	−0.3311	−0.1255	0.7980	0.053*	
C53	−0.5086 (3)	−0.4242 (2)	0.90406 (17)	0.0421 (7)	
C54	−0.5539 (3)	−0.5112 (2)	0.90958 (16)	0.0406 (7)	
C55	−0.4853 (3)	−0.5802 (2)	0.9032 (2)	0.0503 (8)	
H55	−0.4064	−0.5733	0.8939	0.060*	
C56	−0.5330 (4)	−0.6598 (2)	0.9106 (2)	0.0546 (9)	
H56	−0.4862	−0.7065	0.9070	0.065*	
C57	−0.6484 (4)	−0.6697 (2)	0.9231 (2)	0.0564 (10)	
H57	−0.6801	−0.7230	0.9273	0.068*	
C58	−0.7180 (4)	−0.6020 (3)	0.9294 (2)	0.0645 (11)	
H58	−0.7968	−0.6095	0.9381	0.077*	
C59	−0.6714 (3)	−0.5221 (3)	0.9230 (2)	0.0569 (9)	
H59	−0.7188	−0.4761	0.9276	0.068*	
C60	−0.1324 (2)	0.14297 (17)	0.96558 (14)	0.0259 (5)	
C61	−0.2385 (3)	0.1815 (3)	0.96601 (17)	0.0497 (9)	
H61	−0.2750	0.1961	0.9235	0.060*	
C62	−0.2926 (3)	0.1991 (3)	1.02882 (18)	0.0536 (10)	
H62	−0.3642	0.2256	1.0285	0.064*	
C63	−0.2372 (2)	0.17610 (19)	1.09139 (14)	0.0302 (6)	
C64	−0.1345 (3)	0.1347 (2)	1.09347 (15)	0.0357 (6)	
H64	−0.0995	0.1177	1.1368	0.043*	
C65	−0.0820 (2)	0.1179 (2)	1.03035 (15)	0.0351 (6)	
H65	−0.0117	0.0892	1.0317	0.042*	
C66	−0.2559 (3)	0.2544 (2)	1.17485 (17)	0.0384 (7)	
C67	−0.3283 (3)	0.2608 (2)	1.24327 (17)	0.0400 (7)	
C68	−0.4151 (3)	0.2020 (2)	1.28323 (16)	0.0421 (7)	
H68	−0.4283	0.1548	1.2689	0.051*	
C69	−0.4840 (3)	0.2123 (2)	1.34514 (17)	0.0492 (8)	
H69	−0.5436	0.1725	1.3713	0.059*	
C70	−0.4646 (3)	0.2796 (3)	1.3675 (2)	0.0557 (9)	
H70	−0.5107	0.2861	1.4089	0.067*	
C71	−0.3776 (4)	0.3377 (3)	1.3292 (3)	0.0747 (13)	
H71	−0.3635	0.3832	1.3455	0.090*	
C72	−0.3088 (4)	0.3300 (3)	1.2656 (3)	0.0710 (13)	
H72	−0.2508	0.3710	1.2388	0.085*	
C73	−0.0926 (2)	0.15633 (19)	0.60209 (15)	0.0313 (6)	
H73	−0.0385	0.1233	0.5830	0.038*	
C74	−0.1770 (2)	0.19815 (19)	0.56027 (15)	0.0312 (6)	
H74	−0.1802	0.1935	0.5138	0.037*	
C75	−0.2573 (2)	0.24745 (17)	0.58849 (14)	0.0270 (5)	
C76	−0.2488 (3)	0.25382 (19)	0.65738 (16)	0.0352 (6)	
H76	−0.3008	0.2872	0.6775	0.042*	
C77	−0.1611 (3)	0.20943 (19)	0.69524 (15)	0.0337 (6)	
H77	−0.1551	0.2135	0.7415	0.040*	
C78	−0.3504 (2)	0.28922 (18)	0.54773 (15)	0.0305 (6)	
N7A	0.7563 (4)	0.3550 (3)	0.7825 (2)	0.0788 (11)	0.666 (4)
C84A	0.9540 (5)	0.4908 (4)	0.8050 (3)	0.0925 (15)	0.666 (4)
H84A	0.9669	0.5093	0.7524	0.111*	0.666 (4)
C79A	0.8132 (5)	0.3884 (4)	0.8153 (4)	0.0667 (17)	0.666 (4)
C80A	0.8777 (5)	0.4256 (4)	0.8530 (3)	0.0574 (14)	0.666 (4)
C81A	0.8662 (5)	0.3948 (4)	0.9316 (3)	0.0591 (15)	0.666 (4)
H81A	0.8120	0.3507	0.9591	0.071*	0.666 (4)
C82A	0.9266 (4)	0.4238 (3)	0.9694 (2)	0.0357 (10)	0.666 (4)
H82A	0.9196	0.3968	1.0219	0.043*	0.666 (4)
C83A	1.0147 (7)	0.5281 (5)	0.8528 (3)	0.088 (2)	0.666 (4)
H83A	1.0638	0.5763	0.8271	0.106*	0.666 (4)
N8A	1.0013 (5)	0.4952 (4)	0.9309 (3)	0.0880 (19)	0.666 (4)
N7B	0.7563 (4)	0.3550 (3)	0.7825 (2)	0.0788 (11)	0.334 (4)
C84B	0.9540 (5)	0.4908 (4)	0.8050 (3)	0.0925 (15)	0.334 (4)
H84B	0.9100	0.4837	0.8513	0.111*	0.334 (4)
C79B	0.8307 (7)	0.3948 (6)	0.7609 (6)	0.040 (2)	0.334 (4)
C80B	0.9278 (6)	0.4483 (5)	0.7439 (4)	0.040 (2)	0.334 (4)
C81B	0.9994 (8)	0.4646 (7)	0.6751 (5)	0.050 (3)	0.334 (4)
H81B	0.9835	0.4396	0.6412	0.060*	0.334 (4)
C82B	1.0924 (8)	0.5176 (8)	0.6587 (5)	0.067 (3)	0.334 (4)
H82B	1.1398	0.5287	0.6129	0.080*	0.334 (4)
C83B	1.0481 (10)	0.5365 (9)	0.7763 (6)	0.085 (5)	0.334 (4)
H83B	1.0732	0.5615	0.8077	0.101*	0.334 (4)
N8B	1.1183 (9)	0.5547 (9)	0.7068 (7)	0.084 (4)	0.334 (4)

### Atomic displacement parameters (Å^2^)

**Table:**

	*U*^11^	*U*^22^	*U*^33^	*U*^12^	*U*^13^	*U*^23^
Zn	0.02045 (14)	0.02657 (15)	0.01861 (14)	−0.00220 (11)	−0.00024 (10)	−0.01128 (11)
O1	0.0395 (12)	0.0347 (11)	0.0588 (13)	−0.0150 (9)	0.0040 (10)	−0.0255 (10)
O2	0.0441 (13)	0.0425 (12)	0.0554 (13)	−0.0144 (10)	0.0045 (10)	−0.0260 (11)
O3	0.0303 (10)	0.0484 (12)	0.0277 (9)	−0.0014 (9)	0.0008 (8)	−0.0255 (9)
O4	0.0392 (11)	0.0552 (13)	0.0337 (10)	−0.0123 (10)	−0.0009 (9)	−0.0236 (10)
O5	0.0488 (14)	0.0451 (13)	0.0556 (14)	−0.0205 (11)	−0.0048 (11)	−0.0133 (11)
O6	0.0612 (16)	0.0465 (14)	0.0564 (15)	−0.0133 (12)	0.0021 (12)	−0.0215 (12)
O7	0.0407 (11)	0.0425 (11)	0.0265 (9)	0.0017 (9)	0.0049 (8)	−0.0216 (8)
O8	0.0506 (14)	0.0699 (17)	0.0618 (15)	−0.0147 (13)	0.0104 (12)	−0.0425 (14)
N1	0.0225 (10)	0.0279 (11)	0.0231 (10)	−0.0039 (8)	0.0016 (8)	−0.0152 (8)
N2	0.0225 (10)	0.0315 (11)	0.0210 (9)	−0.0017 (8)	−0.0002 (8)	−0.0142 (8)
N3	0.0241 (10)	0.0282 (11)	0.0185 (9)	−0.0020 (8)	−0.0008 (8)	−0.0098 (8)
N4	0.0223 (10)	0.0274 (11)	0.0209 (9)	−0.0008 (8)	−0.0016 (8)	−0.0115 (8)
N5	0.0249 (11)	0.0290 (11)	0.0210 (10)	−0.0018 (9)	−0.0022 (8)	−0.0087 (8)
N6	0.0347 (13)	0.0369 (13)	0.0413 (13)	0.0010 (11)	−0.0129 (11)	−0.0134 (11)
C1	0.0222 (12)	0.0275 (12)	0.0236 (11)	−0.0024 (10)	0.0004 (9)	−0.0122 (10)
C2	0.0274 (13)	0.0320 (14)	0.0280 (12)	−0.0082 (11)	0.0080 (10)	−0.0157 (11)
C3	0.0287 (13)	0.0333 (14)	0.0242 (12)	−0.0051 (11)	0.0058 (10)	−0.0145 (11)
C4	0.0220 (12)	0.0295 (13)	0.0208 (11)	−0.0012 (10)	0.0006 (9)	−0.0127 (10)
C5	0.0223 (12)	0.0309 (13)	0.0203 (11)	0.0008 (10)	−0.0024 (9)	−0.0130 (10)
C6	0.0245 (12)	0.0271 (12)	0.0220 (11)	0.0015 (10)	−0.0044 (9)	−0.0129 (10)
C7	0.0349 (14)	0.0309 (14)	0.0280 (12)	−0.0020 (11)	−0.0037 (11)	−0.0175 (11)
C8	0.0329 (14)	0.0285 (13)	0.0296 (13)	−0.0060 (11)	−0.0028 (11)	−0.0139 (11)
C9	0.0238 (12)	0.0270 (12)	0.0228 (11)	−0.0021 (10)	−0.0056 (9)	−0.0099 (10)
C10	0.0260 (12)	0.0275 (13)	0.0222 (11)	−0.0029 (10)	−0.0056 (10)	−0.0069 (10)
C11	0.0234 (12)	0.0289 (13)	0.0200 (11)	−0.0015 (10)	−0.0010 (9)	−0.0070 (10)
C12	0.0304 (14)	0.0352 (14)	0.0219 (12)	−0.0071 (11)	0.0041 (10)	−0.0072 (10)
C13	0.0289 (13)	0.0376 (15)	0.0208 (11)	−0.0017 (11)	0.0044 (10)	−0.0109 (11)
C14	0.0218 (12)	0.0314 (13)	0.0170 (10)	0.0022 (10)	−0.0017 (9)	−0.0082 (9)
C15	0.0221 (12)	0.0332 (13)	0.0194 (11)	0.0029 (10)	−0.0032 (9)	−0.0125 (10)
C16	0.0219 (12)	0.0357 (14)	0.0233 (11)	0.0014 (10)	−0.0026 (9)	−0.0163 (10)
C17	0.0294 (13)	0.0410 (15)	0.0299 (13)	−0.0022 (11)	−0.0020 (11)	−0.0229 (12)
C18	0.0294 (13)	0.0366 (14)	0.0330 (13)	−0.0041 (11)	−0.0025 (11)	−0.0221 (12)
C19	0.0235 (12)	0.0292 (13)	0.0258 (12)	0.0003 (10)	−0.0029 (10)	−0.0151 (10)
C20	0.0230 (12)	0.0253 (12)	0.0257 (12)	−0.0024 (10)	−0.0010 (9)	−0.0115 (10)
C21	0.0233 (12)	0.0276 (13)	0.0267 (12)	−0.0019 (10)	0.0006 (10)	−0.0146 (10)
C22	0.0332 (14)	0.0288 (13)	0.0301 (13)	−0.0058 (11)	−0.0032 (11)	−0.0100 (11)
C23	0.0294 (14)	0.0388 (15)	0.0373 (15)	−0.0059 (12)	−0.0052 (11)	−0.0186 (12)
C24	0.0305 (14)	0.0293 (14)	0.0435 (15)	−0.0081 (11)	0.0039 (12)	−0.0200 (12)
C25	0.0455 (17)	0.0241 (13)	0.0376 (15)	−0.0029 (12)	−0.0013 (13)	−0.0110 (11)
C26	0.0340 (14)	0.0307 (14)	0.0324 (14)	−0.0020 (11)	−0.0053 (11)	−0.0123 (11)
C27	0.0375 (15)	0.0279 (14)	0.0375 (15)	−0.0087 (12)	−0.0033 (12)	−0.0084 (12)
C28	0.0418 (17)	0.0369 (16)	0.0526 (19)	−0.0152 (14)	−0.0006 (14)	−0.0168 (14)
C29	0.0462 (19)	0.0424 (18)	0.059 (2)	−0.0122 (15)	−0.0067 (16)	−0.0218 (16)
C30	0.0646 (13)	0.0625 (13)	0.0665 (13)	−0.0052 (9)	−0.0099 (9)	−0.0277 (9)
C31	0.0778 (15)	0.0762 (15)	0.0818 (15)	−0.0080 (9)	−0.0104 (9)	−0.0324 (10)
C32	0.0905 (18)	0.0908 (17)	0.0940 (17)	−0.0060 (10)	−0.0093 (10)	−0.0364 (11)
C33	0.0699 (14)	0.0694 (14)	0.0734 (14)	−0.0073 (9)	−0.0060 (9)	−0.0300 (10)
C34	0.0257 (12)	0.0262 (12)	0.0216 (11)	−0.0039 (10)	0.0003 (9)	−0.0124 (9)
C35	0.0319 (14)	0.0458 (16)	0.0281 (13)	0.0122 (12)	−0.0077 (11)	−0.0201 (12)
C36	0.0365 (15)	0.0481 (17)	0.0243 (12)	0.0084 (13)	−0.0079 (11)	−0.0192 (12)
C37	0.0290 (13)	0.0325 (13)	0.0226 (11)	−0.0017 (11)	0.0003 (10)	−0.0172 (10)
C38	0.0329 (14)	0.0423 (16)	0.0302 (13)	0.0118 (12)	−0.0039 (11)	−0.0187 (12)
C39	0.0337 (14)	0.0446 (16)	0.0230 (12)	0.0085 (12)	−0.0072 (11)	−0.0161 (11)
C40	0.0326 (14)	0.0276 (13)	0.0219 (11)	−0.0018 (11)	−0.0016 (10)	−0.0087 (10)
C41	0.0322 (13)	0.0264 (13)	0.0207 (11)	−0.0011 (10)	0.0006 (10)	−0.0083 (10)
C42	0.0275 (13)	0.0440 (16)	0.0317 (14)	−0.0025 (12)	0.0028 (11)	−0.0194 (12)
C43	0.0396 (16)	0.0504 (18)	0.0321 (14)	−0.0048 (14)	−0.0032 (12)	−0.0225 (13)
C44	0.0425 (16)	0.0438 (16)	0.0234 (12)	−0.0011 (13)	0.0038 (11)	−0.0163 (12)
C45	0.0327 (15)	0.0459 (17)	0.0259 (13)	0.0005 (13)	0.0061 (11)	−0.0092 (12)
C46	0.0309 (14)	0.0370 (15)	0.0238 (12)	−0.0022 (11)	−0.0014 (10)	−0.0076 (11)
C47	0.0316 (14)	0.0302 (13)	0.0218 (11)	−0.0064 (11)	−0.0030 (10)	−0.0065 (10)
C48	0.0350 (15)	0.0332 (15)	0.0482 (17)	−0.0037 (12)	−0.0092 (13)	−0.0124 (13)
C49	0.0437 (18)	0.0293 (15)	0.0535 (19)	−0.0054 (13)	−0.0060 (15)	−0.0092 (13)
C50	0.0431 (17)	0.0391 (16)	0.0362 (15)	−0.0175 (13)	−0.0035 (13)	−0.0093 (13)
C51	0.0408 (18)	0.0473 (19)	0.057 (2)	−0.0148 (15)	−0.0201 (16)	0.0016 (16)
C52	0.0375 (16)	0.0365 (16)	0.0474 (18)	−0.0070 (13)	−0.0160 (14)	0.0025 (13)
C53	0.0491 (18)	0.0435 (17)	0.0311 (14)	−0.0138 (15)	−0.0040 (13)	−0.0095 (13)
C54	0.0521 (19)	0.0385 (16)	0.0308 (14)	−0.0159 (14)	−0.0067 (13)	−0.0097 (12)
C55	0.049 (2)	0.049 (2)	0.0509 (19)	−0.0127 (16)	−0.0099 (16)	−0.0130 (16)
C56	0.068 (2)	0.0427 (19)	0.056 (2)	−0.0078 (17)	−0.0140 (18)	−0.0178 (16)
C57	0.077 (3)	0.046 (2)	0.0474 (19)	−0.0231 (19)	−0.0098 (18)	−0.0156 (16)
C58	0.051 (2)	0.072 (3)	0.073 (3)	−0.030 (2)	0.0084 (19)	−0.033 (2)
C59	0.054 (2)	0.054 (2)	0.064 (2)	−0.0143 (17)	0.0069 (18)	−0.0286 (18)
C60	0.0249 (12)	0.0333 (13)	0.0218 (11)	0.0006 (10)	0.0009 (9)	−0.0144 (10)
C61	0.0401 (17)	0.087 (3)	0.0298 (15)	0.0263 (17)	−0.0125 (13)	−0.0308 (16)
C62	0.0407 (18)	0.091 (3)	0.0364 (16)	0.0330 (18)	−0.0110 (14)	−0.0339 (18)
C63	0.0333 (14)	0.0372 (15)	0.0227 (12)	0.0006 (11)	0.0031 (10)	−0.0167 (11)
C64	0.0385 (15)	0.0495 (17)	0.0237 (12)	0.0112 (13)	−0.0078 (11)	−0.0189 (12)
C65	0.0325 (14)	0.0500 (17)	0.0276 (13)	0.0164 (13)	−0.0082 (11)	−0.0204 (12)
C66	0.0389 (16)	0.0473 (17)	0.0350 (15)	0.0039 (14)	−0.0037 (13)	−0.0234 (13)
C67	0.0428 (17)	0.0503 (18)	0.0373 (15)	0.0135 (14)	−0.0097 (13)	−0.0284 (14)
C68	0.058 (2)	0.0424 (17)	0.0270 (14)	0.0111 (15)	−0.0031 (13)	−0.0165 (12)
C69	0.058 (2)	0.059 (2)	0.0285 (15)	0.0131 (17)	0.0002 (14)	−0.0174 (14)
C70	0.057 (2)	0.081 (3)	0.0406 (18)	0.013 (2)	−0.0009 (16)	−0.0400 (19)
C71	0.082 (3)	0.092 (3)	0.081 (3)	−0.005 (3)	0.004 (2)	−0.073 (3)
C72	0.065 (3)	0.090 (3)	0.082 (3)	−0.018 (2)	0.014 (2)	−0.066 (3)
C73	0.0283 (13)	0.0413 (15)	0.0292 (13)	0.0026 (11)	−0.0018 (10)	−0.0199 (12)
C74	0.0328 (14)	0.0410 (15)	0.0237 (12)	0.0005 (12)	−0.0051 (10)	−0.0161 (11)
C75	0.0259 (13)	0.0257 (13)	0.0272 (12)	−0.0026 (10)	−0.0039 (10)	−0.0067 (10)
C76	0.0418 (16)	0.0355 (15)	0.0314 (14)	0.0117 (12)	−0.0072 (12)	−0.0168 (12)
C77	0.0420 (16)	0.0356 (15)	0.0267 (13)	0.0074 (12)	−0.0068 (11)	−0.0156 (11)
C78	0.0315 (14)	0.0291 (13)	0.0312 (13)	−0.0034 (11)	−0.0042 (11)	−0.0111 (11)
N7A	0.090 (3)	0.087 (3)	0.073 (3)	0.026 (2)	−0.023 (2)	−0.044 (2)
C84A	0.0921 (16)	0.0923 (16)	0.0925 (16)	0.0005 (4)	−0.0132 (4)	−0.0337 (6)
C79A	0.068 (4)	0.064 (4)	0.070 (5)	0.016 (3)	−0.010 (3)	−0.030 (3)
C80A	0.0572 (15)	0.0570 (15)	0.0577 (15)	0.0006 (5)	−0.0081 (5)	−0.0211 (7)
C81A	0.0590 (15)	0.0586 (15)	0.0594 (15)	0.0005 (5)	−0.0082 (5)	−0.0217 (7)
C82A	0.0360 (11)	0.0361 (11)	0.0358 (11)	−0.0003 (5)	−0.0057 (5)	−0.0140 (6)
C83A	0.088 (2)	0.088 (2)	0.089 (2)	0.0000 (5)	−0.0125 (6)	−0.0325 (9)
N8A	0.0878 (19)	0.0880 (19)	0.0884 (19)	0.0007 (5)	−0.0131 (6)	−0.0327 (8)
N7B	0.090 (3)	0.087 (3)	0.073 (3)	0.026 (2)	−0.023 (2)	−0.044 (2)
C84B	0.0921 (16)	0.0923 (16)	0.0925 (16)	0.0005 (4)	−0.0132 (4)	−0.0337 (6)
C79B	0.042 (5)	0.050 (6)	0.039 (5)	0.015 (4)	−0.018 (4)	−0.027 (5)
C80B	0.043 (5)	0.037 (5)	0.052 (5)	0.014 (4)	−0.021 (4)	−0.028 (4)
C81B	0.054 (6)	0.049 (6)	0.057 (6)	0.018 (5)	−0.032 (5)	−0.024 (5)
C82B	0.057 (7)	0.068 (8)	0.081 (9)	0.019 (6)	−0.013 (6)	−0.035 (7)
C83B	0.070 (9)	0.100 (11)	0.123 (13)	−0.009 (8)	−0.040 (9)	−0.075 (10)
N8B	0.061 (7)	0.103 (10)	0.116 (10)	−0.012 (6)	−0.017 (7)	−0.070 (9)

### Geometric parameters (Å, º)

**Table:**

Zn—N1	2.053 (2)	C38—C39	1.389 (4)
Zn—N2	2.060 (2)	C38—H38	0.9300
Zn—N3	2.060 (2)	C39—H39	0.9300
Zn—N4	2.068 (2)	C40—C41	1.486 (3)
Zn—N5	2.159 (2)	C41—C42	1.386 (4)
O1—C27	1.342 (4)	C41—C46	1.394 (4)
O1—C24	1.416 (3)	C42—C43	1.388 (4)
O2—C27	1.198 (3)	C42—H42	0.9300
O3—C40	1.361 (3)	C43—C44	1.377 (4)
O3—C37	1.409 (3)	C43—H43	0.9300
O4—C40	1.194 (3)	C44—C45	1.378 (4)
O5—C53	1.351 (4)	C44—H44	0.9300
O5—C50	1.413 (3)	C45—C46	1.386 (4)
O6—C53	1.199 (4)	C45—H45	0.9300
O7—C66	1.358 (3)	C46—H46	0.9300
O7—C63	1.405 (3)	C47—C48	1.382 (4)
O8—C66	1.191 (4)	C47—C52	1.386 (4)
N1—C1	1.367 (3)	C48—C49	1.389 (4)
N1—C4	1.368 (3)	C48—H48	0.9300
N2—C19	1.368 (3)	C49—C50	1.370 (5)
N2—C16	1.373 (3)	C49—H49	0.9300
N3—C11	1.367 (3)	C50—C51	1.381 (5)
N3—C14	1.371 (3)	C51—C52	1.384 (4)
N4—C6	1.370 (3)	C51—H51	0.9300
N4—C9	1.373 (3)	C52—H52	0.9300
N5—C77	1.327 (3)	C53—C54	1.494 (4)
N5—C73	1.334 (3)	C54—C55	1.375 (5)
N6—C78	1.138 (4)	C54—C59	1.386 (5)
C1—C20	1.406 (3)	C55—C56	1.384 (5)
C1—C2	1.445 (3)	C55—H55	0.9300
C2—C3	1.348 (3)	C56—C57	1.359 (5)
C2—H2	0.9300	C56—H56	0.9300
C3—C4	1.440 (3)	C57—C58	1.365 (6)
C3—H3	0.9300	C57—H57	0.9300
C4—C5	1.396 (3)	C58—C59	1.388 (5)
C5—C6	1.405 (3)	C58—H58	0.9300
C5—C34	1.501 (3)	C59—H59	0.9300
C6—C7	1.443 (3)	C60—C61	1.373 (4)
C7—C8	1.352 (4)	C60—C65	1.379 (4)
C7—H7	0.9300	C61—C62	1.394 (4)
C8—C9	1.446 (3)	C61—H61	0.9300
C8—H8	0.9300	C62—C63	1.380 (4)
C9—C10	1.405 (3)	C62—H62	0.9300
C10—C11	1.406 (3)	C63—C64	1.356 (4)
C10—C47	1.495 (3)	C64—C65	1.387 (4)
C11—C12	1.449 (3)	C64—H64	0.9300
C12—C13	1.355 (4)	C65—H65	0.9300
C12—H12	0.9300	C66—C67	1.490 (4)
C13—C14	1.439 (3)	C67—C68	1.368 (5)
C13—H13	0.9300	C67—C72	1.381 (5)
C14—C15	1.406 (3)	C68—C69	1.393 (4)
C15—C16	1.401 (3)	C68—H68	0.9300
C15—C60	1.504 (3)	C69—C70	1.350 (5)
C16—C17	1.446 (4)	C69—H69	0.9300
C17—C18	1.349 (4)	C70—C71	1.358 (6)
C17—H17	0.9300	C70—H70	0.9300
C18—C19	1.447 (3)	C71—C72	1.403 (5)
C18—H18	0.9300	C71—H71	0.9300
C19—C20	1.409 (3)	C72—H72	0.9300
C20—C21	1.502 (3)	C73—C74	1.370 (4)
C21—C26	1.388 (4)	C73—H73	0.9300
C21—C22	1.388 (4)	C74—C75	1.384 (4)
C22—C23	1.386 (4)	C74—H74	0.9300
C22—H22	0.9300	C75—C76	1.385 (4)
C23—C24	1.366 (4)	C75—C78	1.442 (4)
C23—H23	0.9300	C76—C77	1.375 (4)
C24—C25	1.375 (4)	C76—H76	0.9300
C25—C26	1.391 (4)	C77—H77	0.9300
C25—H25	0.9300	N7A—C79A	1.254 (6)
C26—H26	0.9300	C84A—C80A	1.380 (8)
C27—C28	1.486 (4)	C84A—C83A	1.547 (9)
C28—C29	1.378 (5)	C84A—H84A	0.9300
C28—C33	1.379 (5)	C79A—C80A	1.419 (4)
C29—C30	1.387 (5)	C80A—C81A	1.391 (7)
C29—H29	0.9300	C81A—C82A	1.309 (7)
C30—C31	1.365 (6)	C81A—H81A	0.9300
C30—H30	0.9300	C82A—N8A	1.392 (4)
C31—C32	1.381 (7)	C82A—H82A	0.9300
C31—H31	0.9300	C83A—N8A	1.378 (4)
C32—C33	1.388 (7)	C83A—H83A	0.9300
C32—H32	0.9300	C79B—C80B	1.402 (5)
C33—H33	0.9300	C80B—C81B	1.398 (5)
C34—C35	1.384 (4)	C81B—C82B	1.358 (12)
C34—C39	1.384 (4)	C81B—H81B	0.9300
C35—C36	1.394 (4)	C82B—N8B	1.352 (12)
C35—H35	0.9300	C82B—H82B	0.9300
C36—C37	1.371 (4)	C83B—N8B	1.398 (5)
C36—H36	0.9300	C83B—H83B	0.9300
C37—C38	1.376 (4)		
			
N1—Zn—N2	89.25 (8)	C34—C39—H39	119.3
N1—Zn—N3	167.78 (8)	C38—C39—H39	119.3
N2—Zn—N3	89.16 (8)	O4—C40—O3	123.7 (2)
N1—Zn—N4	89.14 (8)	O4—C40—C41	124.9 (2)
N2—Zn—N4	160.84 (8)	O3—C40—C41	111.4 (2)
N3—Zn—N4	88.39 (8)	C42—C41—C46	120.2 (2)
N1—Zn—N5	96.43 (8)	C42—C41—C40	122.5 (2)
N2—Zn—N5	100.65 (8)	C46—C41—C40	117.2 (2)
N3—Zn—N5	95.77 (8)	C41—C42—C43	119.4 (3)
N4—Zn—N5	98.50 (8)	C41—C42—H42	120.3
C27—O1—C24	117.7 (2)	C43—C42—H42	120.3
C40—O3—C37	119.1 (2)	C44—C43—C42	120.3 (3)
C53—O5—C50	121.6 (3)	C44—C43—H43	119.8
C66—O7—C63	117.6 (2)	C42—C43—H43	119.8
C1—N1—C4	106.66 (19)	C43—C44—C45	120.5 (3)
C1—N1—Zn	126.96 (15)	C43—C44—H44	119.7
C4—N1—Zn	125.78 (16)	C45—C44—H44	119.7
C19—N2—C16	106.7 (2)	C44—C45—C46	120.0 (3)
C19—N2—Zn	126.39 (15)	C44—C45—H45	120.0
C16—N2—Zn	126.62 (17)	C46—C45—H45	120.0
C11—N3—C14	106.86 (19)	C45—C46—C41	119.6 (3)
C11—N3—Zn	127.11 (16)	C45—C46—H46	120.2
C14—N3—Zn	125.72 (16)	C41—C46—H46	120.2
C6—N4—C9	106.7 (2)	C48—C47—C52	118.0 (3)
C6—N4—Zn	125.86 (16)	C48—C47—C10	122.1 (2)
C9—N4—Zn	127.11 (16)	C52—C47—C10	119.9 (2)
C77—N5—C73	118.3 (2)	C47—C48—C49	120.6 (3)
C77—N5—Zn	120.75 (17)	C47—C48—H48	119.7
C73—N5—Zn	120.78 (18)	C49—C48—H48	119.7
N1—C1—C20	125.2 (2)	C50—C49—C48	120.0 (3)
N1—C1—C2	109.7 (2)	C50—C49—H49	120.0
C20—C1—C2	125.0 (2)	C48—C49—H49	120.0
C3—C2—C1	106.7 (2)	C49—C50—C51	120.7 (3)
C3—C2—H2	126.7	C49—C50—O5	115.1 (3)
C1—C2—H2	126.7	C51—C50—O5	124.0 (3)
C2—C3—C4	107.4 (2)	C50—C51—C52	118.5 (3)
C2—C3—H3	126.3	C50—C51—H51	120.7
C4—C3—H3	126.3	C52—C51—H51	120.7
N1—C4—C5	125.3 (2)	C51—C52—C47	122.0 (3)
N1—C4—C3	109.5 (2)	C51—C52—H52	119.0
C5—C4—C3	124.9 (2)	C47—C52—H52	119.0
C4—C5—C6	125.7 (2)	O6—C53—O5	124.2 (3)
C4—C5—C34	116.6 (2)	O6—C53—C54	125.1 (3)
C6—C5—C34	117.7 (2)	O5—C53—C54	110.8 (3)
N4—C6—C5	125.4 (2)	C55—C54—C59	119.2 (3)
N4—C6—C7	109.6 (2)	C55—C54—C53	123.4 (3)
C5—C6—C7	124.9 (2)	C59—C54—C53	117.3 (3)
C8—C7—C6	107.1 (2)	C54—C55—C56	120.4 (3)
C8—C7—H7	126.5	C54—C55—H55	119.8
C6—C7—H7	126.5	C56—C55—H55	119.8
C7—C8—C9	107.1 (2)	C57—C56—C55	120.0 (4)
C7—C8—H8	126.5	C57—C56—H56	120.0
C9—C8—H8	126.5	C55—C56—H56	120.0
N4—C9—C10	125.8 (2)	C56—C57—C58	120.5 (3)
N4—C9—C8	109.4 (2)	C56—C57—H57	119.7
C10—C9—C8	124.8 (2)	C58—C57—H57	119.7
C9—C10—C11	124.6 (2)	C57—C58—C59	120.1 (4)
C9—C10—C47	117.7 (2)	C57—C58—H58	119.9
C11—C10—C47	117.7 (2)	C59—C58—H58	119.9
N3—C11—C10	125.9 (2)	C54—C59—C58	119.7 (4)
N3—C11—C12	109.6 (2)	C54—C59—H59	120.2
C10—C11—C12	124.4 (2)	C58—C59—H59	120.2
C13—C12—C11	106.6 (2)	C61—C60—C65	118.5 (2)
C13—C12—H12	126.7	C61—C60—C15	121.1 (2)
C11—C12—H12	126.7	C65—C60—C15	120.3 (2)
C12—C13—C14	107.4 (2)	C60—C61—C62	121.4 (3)
C12—C13—H13	126.3	C60—C61—H61	119.3
C14—C13—H13	126.3	C62—C61—H61	119.3
N3—C14—C15	126.0 (2)	C63—C62—C61	118.3 (3)
N3—C14—C13	109.5 (2)	C63—C62—H62	120.9
C15—C14—C13	124.4 (2)	C61—C62—H62	120.9
C16—C15—C14	125.2 (2)	C64—C63—C62	121.4 (2)
C16—C15—C60	117.9 (2)	C64—C63—O7	120.5 (2)
C14—C15—C60	116.9 (2)	C62—C63—O7	117.8 (2)
N2—C16—C15	125.4 (2)	C63—C64—C65	119.4 (3)
N2—C16—C17	109.6 (2)	C63—C64—H64	120.3
C15—C16—C17	125.0 (2)	C65—C64—H64	120.3
C18—C17—C16	107.0 (2)	C60—C65—C64	121.0 (3)
C18—C17—H17	126.5	C60—C65—H65	119.5
C16—C17—H17	126.5	C64—C65—H65	119.5
C17—C18—C19	107.2 (2)	O8—C66—O7	123.3 (3)
C17—C18—H18	126.4	O8—C66—C67	126.1 (3)
C19—C18—H18	126.4	O7—C66—C67	110.6 (3)
N2—C19—C20	126.1 (2)	C68—C67—C72	119.1 (3)
N2—C19—C18	109.5 (2)	C68—C67—C66	122.3 (3)
C20—C19—C18	124.4 (2)	C72—C67—C66	118.5 (3)
C1—C20—C19	125.0 (2)	C67—C68—C69	120.6 (3)
C1—C20—C21	117.7 (2)	C67—C68—H68	119.7
C19—C20—C21	117.2 (2)	C69—C68—H68	119.7
C26—C21—C22	118.6 (2)	C70—C69—C68	120.4 (4)
C26—C21—C20	122.4 (2)	C70—C69—H69	119.8
C22—C21—C20	118.9 (2)	C68—C69—H69	119.8
C23—C22—C21	120.9 (3)	C69—C70—C71	119.8 (3)
C23—C22—H22	119.5	C69—C70—H70	120.1
C21—C22—H22	119.5	C71—C70—H70	120.1
C24—C23—C22	118.8 (3)	C70—C71—C72	120.8 (3)
C24—C23—H23	120.6	C70—C71—H71	119.6
C22—C23—H23	120.6	C72—C71—H71	119.6
C23—C24—C25	122.5 (3)	C67—C72—C71	119.2 (4)
C23—C24—O1	117.9 (3)	C67—C72—H72	120.4
C25—C24—O1	119.5 (3)	C71—C72—H72	120.4
C24—C25—C26	118.1 (3)	N5—C73—C74	122.7 (2)
C24—C25—H25	120.9	N5—C73—H73	118.7
C26—C25—H25	120.9	C74—C73—H73	118.7
C21—C26—C25	121.1 (3)	C73—C74—C75	118.7 (2)
C21—C26—H26	119.4	C73—C74—H74	120.6
C25—C26—H26	119.4	C75—C74—H74	120.6
O2—C27—O1	123.5 (3)	C74—C75—C76	119.0 (2)
O2—C27—C28	124.9 (3)	C74—C75—C78	120.3 (2)
O1—C27—C28	111.6 (3)	C76—C75—C78	120.7 (2)
C29—C28—C33	120.2 (3)	C77—C76—C75	118.1 (3)
C29—C28—C27	122.8 (3)	C77—C76—H76	121.0
C33—C28—C27	117.1 (3)	C75—C76—H76	121.0
C28—C29—C30	120.0 (3)	N5—C77—C76	123.3 (2)
C28—C29—H29	120.0	N5—C77—H77	118.4
C30—C29—H29	120.0	C76—C77—H77	118.4
C31—C30—C29	119.7 (4)	N6—C78—C75	177.9 (3)
C31—C30—H30	120.1	C80A—C84A—C83A	108.8 (5)
C29—C30—H30	120.1	C80A—C84A—H84A	125.6
C30—C31—C32	120.7 (5)	C83A—C84A—H84A	125.6
C30—C31—H31	119.6	N7A—C79A—C80A	179.5 (7)
C32—C31—H31	119.6	C84A—C80A—C81A	124.3 (5)
C31—C32—C33	119.6 (5)	C84A—C80A—C79A	114.1 (5)
C31—C32—H32	120.2	C81A—C80A—C79A	121.5 (6)
C33—C32—H32	120.2	C82A—C81A—C80A	124.3 (5)
C28—C33—C32	119.7 (4)	C82A—C81A—H81A	117.9
C28—C33—H33	120.2	C80A—C81A—H81A	117.9
C32—C33—H33	120.2	C81A—C82A—N8A	119.8 (4)
C35—C34—C39	118.7 (2)	C81A—C82A—H82A	120.1
C35—C34—C5	120.5 (2)	N8A—C82A—H82A	120.1
C39—C34—C5	120.8 (2)	N8A—C83A—C84A	124.8 (5)
C34—C35—C36	120.6 (2)	N8A—C83A—H83A	117.6
C34—C35—H35	119.7	C84A—C83A—H83A	117.6
C36—C35—H35	119.7	C83A—N8A—C82A	117.6 (4)
C37—C36—C35	119.2 (2)	C81B—C80B—C79B	119.3 (8)
C37—C36—H36	120.4	C82B—C81B—C80B	118.8 (8)
C35—C36—H36	120.4	C82B—C81B—H81B	120.6
C36—C37—C38	121.6 (2)	C80B—C81B—H81B	120.6
C36—C37—O3	115.9 (2)	N8B—C82B—C81B	122.3 (5)
C38—C37—O3	122.2 (2)	N8B—C82B—H82B	118.9
C37—C38—C39	118.5 (2)	C81B—C82B—H82B	118.9
C37—C38—H38	120.7	N8B—C83B—H83B	114.2
C39—C38—H38	120.7	C82B—N8B—C83B	118.7 (8)
C34—C39—C38	121.4 (2)		

### Hydrogen-bond geometry (Å, º)

Cg3, Cg13, Cg18 are the centroids of the N3/C11–C14, C41–C46 and N8*A*–C82*A*–C81*A*–C80*A*–C84*A*–C83*A* rings, respectively.

**Table:**

*D*—H···*A*	*D*—H	H···*A*	*D*···*A*	*D*—H···*A*
C2—H2···N6^i^	0.93	2.45	3.284 (4)	149
C25—H25···N6^ii^	0.93	2.52	3.393 (4)	157
C68—H68···O4^iii^	0.93	2.41	3.150 (4)	136
C72—H72···N8*B*^iv^	0.93	2.58	3.226 (15)	127
C82*A*—H82*A*···O8^i^	0.93	2.38	3.226 (5)	152
C22—H22···*Cg*13^v^	0.93	2.82	3.650 (3)	150
C49—H49···*Cg*18^vi^	0.93	2.61	3.448 (4)	151
C65—H65···*Cg*3^vii^	0.93	2.65	3.457 (4)	145

Symmetry codes: (i) *x*+1, *y*, *z*; (ii) −*x*, −*y*+1, −*z*+1; (iii) *x*−1, *y*, *z*+1; (iv) −*x*+1, −*y*+1, −*z*+2; (v) −*x*+1, −*y*, −*z*+1; (vi) *x*−1, *y*−1, *z*; (vii) −*x*, −*y*, −*z*+2.
